# Human immunodeficiency virus (HIV) type 1 genetic diversity in HIV positive individuals on antiretroviral therapy in a cross-sectional study conducted in Teso, Western Kenya

**DOI:** 10.11604/pamj.2021.38.335.26357

**Published:** 2021-04-07

**Authors:** Maureen Adhiambo, Olipher Makwaga, Ferdinard Adungo, Humphrey Kimani, David Hughes Mulama, Jackson Cheruiyot Korir, Matilu Mwau

**Affiliations:** 1 Department of Biological Sciences, Masinde Muliro University of Science and Technology, Kakamega, Kenya,; 2 Department of Infectious Diseases Control Research, Kenya Medical Research Institute, Nairobi, Kenya

**Keywords:** HIV-1, subtypes, recombinants, phylogenetic

## Abstract

**Introduction:**

high HIV-1 infection rates and genetic diversity especially in African population pose significant challenges in HIV-1 clinical management and drug design and development. HIV-1 is a major health challenge in Kenya and causes mortality and morbidity in the country as well as straining the healthcare system and the economy. This study sought to identify HIV-1 genetic subtypes circulating in Teso, Western Kenya which borders the Republic of Uganda.

**Methods:**

a cross-sectional study was conducted in January 2019 to December 2019. Sequencing of the partial pol gene was carried out on 80 HIV positive individuals on antiretroviral therapy. Subtypes and recombinant forms were generated using the jumping profile hidden Markov model. Alignment of the sequences was done using ClustalW program and phylogenetic tree constructed using MEGA7 neighbor-joining method.

**Results:**

sixty three samples were successful sequenced. In the analysis of these sequences, it was observed that HIV-1 subtype A1 was predominant 43 (68.3%) followed by D 8 (12.7%) and 1 (1.6%) each of C, G and B and inter-subtype recombinants A1-D 3 (4.8%), A1-B 2 (3.2%) and 1 (1.6%) each of A1-A2, A1-C, BC and BD. Phylogenetic analysis of these sequences showed close clustering of closely related and unrelated sequences with reference sequences.

**Conclusion:**

there was observed increased genetic diversity of HIV-1 subtypes which not only pose a challenge in disease control and management but also drug design and development. Therefore, there is need for continued surveillance to enhance future understanding of the geographical distribution and transmission patterns of the HIV epidemic.

## Introduction

Human immunodeficiency virus (HIV) and many simian immunodeficiency viruses (SIV) which infect different African continent´s primate species belong to the family of *retroviridae* and genus lentivirus [1,2]. HIV attacks and destroys the immune system´s CD4 cells which are the major infection fighting cells in the body. Reduction in the number of CD4 cells makes it hard for the body to fight off infections. Failure of treatment, HIV may slowly destroy the body immune system and advance to Acquired Immunodeficiency Syndrome (AIDS) which is a clinical condition that is characterized by immune system failure causing opportunistic infections. Globally, HIV prevalence and deaths related to AIDS has slowly declined over the years. This has been attributed to the life-saving antiretroviral therapy [3]. HIV is categorized into HIV type 1 (HIV-1) and HIV type 2 (HIV-2) on the basis of differences in the viral antigens and genetic characteristics [2]. HIV-1 is more virulent and the major cause for the global AIDS pandemic [1] while HIV-2 is less virulent and less common and its clinical findings are related to HIV-1 [4]. Notably, most HIV-2 cases have been reported in West Africa countries and has expanded in countries with historical and socio-economic ties to this region [5]. Phylogenetic analysis of various HIV-1 strains worldwide has showed three groups, which are M, N and O [6,7]. The global HIV pandemic is mainly caused by group M viruses that are further subdivided into subtypes (A, B, C, D, F, G, H, J and K) and sub-subtypes (A1, A2, A3 and F1, F2). Subtype diversity is influenced by multiple factors, including cross-border demographic patterns that affect transmission and gene flow [8]. The virus subtypes are geographically distributed [8]; subtype G is found in Africa and Central Europe, subtype D is found in Eastern and Central Africa, subtype A in East and West Africa, subtype B is found in Europe, Japan, America, Thailand and Australia while subtype C is found in East Africa, Southern Africa and parts of China [8,9].

HIV-1 subtypes have differing outcomes on rates of transmission, disease progression and development of drug resistance [4]. Rapid disease progression shown by decrease in CD4 cells and high viral loads was identified in subtype C from research studies done in Nairobi [10]. Mother to child transmission of this subtype is very easy [11] and it exhibits increased vaginal shedding than subtypes A and D [12]. Faster disease progression was shown in individuals infected with subtype D as revealed in studies done in Rakai, Uganda while infections with subtype A show slower disease progression [13,14]. Nevertheless, there is no understanding of the clinical implications of each of the HIV-1 subtypes.

The existence of genetic variation in HIV-1 is caused by error-prone reverse transcriptase enzyme, recombination events during replication of the virus, HIV-1 rapid turnover in the body and immune system selective pressures [15]. Recombination of HIV-1 is a complex mechanism that the virus uses for survival to escape the body´s defense mechanism [16]. HIV-1 genetic variability is the major obstacle in the treatment of HIV and development of effective drugs. This variability occurs rapidly with some variants believed to be more virulent and resistant to different antiretroviral drugs [4,6]. The increased genetic variation of HIV-1 presents clinical and public health challenges [17,18]. Precisely, patients monitoring, treatment, diagnostic testing, epidemiologic surveillance and drug development are influenced by HIV-1 genetic diversity [17]. Management of HIV-1 infections has been achieved with highly active antiretroviral therapy; the nucleoside and non-nucleoside reverse transcriptase inhibitors and protease inhibitors. In Kenya, there has been increased scale up of antiretroviral therapy. With the scale up, it´s expected that drug resistant strains will increase among ART exposed individuals and there will be subsequent increase in drug resistance mutations [19].

Past genetic diversity studies conducted in Western and other parts of Kenya have identified differing subtypes and recombinants. In Western Kenya, analysis of HIV pol region of 75 samples identified A1, D, G, C, A2, B, A1-C and A1-D [20]. Another study done on the gag and env regions of HIV-1 in 74 samples revealed A1, C, D, G, A1/C, A1/D, C/A1, D/A1, A1/A2 and A2/C [21]. In Kisumu, analysis of 100 samples identified the following subtypes; A, D, C, G, AD, AC, AG [22]. In Nairobi A1, D, C, G, AD, AC, AG and A2D were identified in analysis of 140 samples [23]. Another study done in Central Kenya identified A1, D and C subtypes [24]. Studies conducted in Western Kenya, which the study region lies were done over a decade ago and due to increased genetic diversity of HIV due to its variability we expect to find new strains of the virus in the population. The study region lies in the Kenya - Uganda border and has two border posts (Busia and Malaba) that are entry points for travelers and truck drivers that go as far as Uganda, Rwanda and Congo. Through this region new strains from these countries are introduced into the Kenyan population.

The new strains identified may confer resistance to antiretroviral drugs making it difficult to manage the HIV epidemic in the country. Current data on HIV-1 genetic diversity is missing in this border region that is characterized by cross-border movement of people. Therefore, this study was aimed at identifying the various HIV-1 subtypes circulating in the Teso region of Western Kenya. The study findings will provide information on the genetic diversity of HIV in this border region of Kenya. The study findings will also provide useful insights to the natural history of the HIV virus at the molecular, host and community levels, which is critical for assessment of the effectiveness of treatment, intervention strategies and informing public health policy.

## Methods

**Study design:** a cross-sectional study was conducted in January 2019 to December 2019 on HIV-1 positive individuals seeking care and treatment at comprehensive care clinics across the health care facilities in Teso region, Western Kenya. These health care facilities participate in viral load testing at the Kenya Medical Research Institute (KEMRI) Alupe Molecular Laboratory.

**Study area:** the study area is a border region with two border points Busia and Malaba which are transit points for travelers and truck drivers travelling as far as East, West and Central Africa. These travels are mainly due to business activities, tourism, better lifestyles and inter-marriages. The presence of some cultural practices, high poverty levels in this region encourage spread of HIV by encouraging indulgence in promiscuous sexual behavior such as early teenage pregnancies, prostitution and wife inheritance. The region is located in Busia county with HIV prevalence rate of approximately 7.7% which is above the Kenyan national HIV prevalence rate which stands at 4.9% [25].

**Study population:** those recruited in the study were HIV positive individuals one year and above whose viral load copies were exceeding 1000 copies/ml after routine viral load testing. Participant´s demographic data on age, gender and ART treatment regimen and current viral load copies were obtained.

**Sample size determination:** statistically significant sample size was obtained using the formula [26]. The Kenya national HIV prevalence rate was used.

$$n_{0}=\frac{Z^{2}pq}{e^{2}}$$

Where n_0_ is the minimum sample size; Z is the confidence level (standard deviation value of 1.96); p is the prevalence of infection =0.049. The prevalence HIV-1 infection in Kenya is (4.9%) [27]; q= (1-p); e is the desired level of precision (5%). Therefore, the sample size was 72 which was rounded to 80.

**Sample collection:** whole blood samples were obtained from study participants who were on antiretroviral therapy (ART) and collected in plasma preparation tubes (Becton Dickinson, San Jose, CA, USA). The samples were centrifuged at 3500 rpm. The obtained plasma was aliquoted into cryotubes and stored at -80 degrees celsius prior to ribonucleic acid (RNA) extraction.

**Laboratory procedures:** RNA extraction and quantification were done using Abbott Molecular m2000 RealTime assays (Abbott Molecular Inc. USA) and Cobas Ampliprep/Cobas Taqman HIV-1 test v.2.0 (Roche Diagnostics, USA) as per the manufacturer´s protocol. The RNA of residual plasma samples with viral load copies greater than 1000 copies/ml of blood was extracted using the Abbott mSample preparation system (Abbott Molecular Inc. USA) as per the manufacturer´s protocol. The Veriti™ 96 well thermal cycler (applied biosystems) was used to transcribe RNA into cDNA using an in-house reverse transcriptase polymerase chain reaction (RT-PCR) protocol. The HIV-1 genotyping kit: amplification module (Thermo Fisher Scientific Inc.) was used. RT-PCR reaction mix which consisted of the RT-PCR master mix and superscript™ III enzyme was prepared for the required number of reactions. The extracted RNA was denatured at 65°C for 10 minutes. The cycling conditions for RT-PCR were: one cycle of reverse transcription at 50°C for 45 minutes, one cycle of enzyme inactivation at 94°C for 2 minutes, 40 cycles of denaturation at 94°C for 15 seconds, 40 cycles of annealing at 50°C for 20 seconds, 40 cycles of extension at 72°C for 2 minutes and one cycle of final extension at 72°C for 10 minutes. Nested PCR was done using the HIV-1 genotyping kit: amplification module (Thermo Fisher Scientific Inc.). Nested PCR master mix and AmpliTaq Gold LD DNA polymerase enzyme was prepared for the required number of reactions. The nested PCR was done under the following cycling conditions: one cycle of initial denaturation at 94°C for 4 minutes, 40 cycles of denaturation at 94°C for 15 seconds, 40 cycles of annealing at 55°C for 20 seconds, 40 cycles of extension at 72°C for 2 minutes and one cycle of final extension at 72°C for 10 minutes. Agarose gel (1%) was used to confirm the PCR amplification success. The gel was visualized on the imaging system and photographed (VisiDoc-It™ Imaging System, CA and USA). Purification of the PCR products was performed using CleanSweep purification reagent (Thermo Fisher Inc.). ExoSAP-IT™ PCR product cleanup reagent was added to 14 μl of each nested PCR product. The plate was vortexed for 2-3 seconds, then centrifuged at 1000 x g for 5-10 seconds. The reaction was purified in the thermocycler under the following cycling conditions: digest at 37°C for 15 minutes and heat deactivation at 80°C for 15 minutes. Cycle sequencing was done using HIV-1 genotyping kit: cycle sequencing module containing HIV-1 sequencing mix (forward 1, forward 2, forward 3, reverse 1, reverse 2, and reverse 3) and pGEM sequencing control (Thermo Fisher Inc.). The cycle sequencing conditions was set at 25 cycles of denaturation, annealing and extension at 96°C for 10 seconds, 50°C for 5 seconds and 60°C for 4 minutes respectively. Purification of cycle sequencing products was done using BigDye XTerminator™ purification kit (Thermo Fisher Inc.) which composed of SAM™ solution and XTerminator™ Solution as per the manufacturer´s instructions.

**Sequencing and phylogenetic analysis:** using the 3730XL DNA analyzer (Applied Biosytems), the HIV-1 pol gene encoding protease and reverse transcriptase (codons 1-999) was directly sequenced. Base calling was facilitated by SeqScanner v.6 (Applied Biosystems Inc. USA) by assessing technical parameters. RECall software was used for sequence editing and generating the consensus sequences. BLAST analysis of the sequences was done to determine library sequences that resembled the query sequence above a certain threshold. HIV-1 genetic variants were obtained with the jumping profile hidden Markov model. For phylogenetic analysis, HIV-1 reference sequences were obtained from the Los Alamos HIV database and the sequences aligned by ClustalW with the generated sequences from the study. Phylogenetic tree was drawn using MEGA7 neighbor-joining method [28-30].

**Statistical analysis:** Stata/SE 13.0 for Windows (StataCorp Texas, USA) was used to analyze the data. Frequencies and percentages were used to express categorical variables. Chi-square χ^2^ test was used to compare two variables example HIV-1 subtypes with age, gender, ART regimen line and viral loads. P-value <0.05 was considered statistically significant.

**Ethical approval:** ethical approval to conduct the study was sought from the Kenya Medical Research Institute Scientific and Ethical Review Unit approval number: KEMRI/SERU/CIPDCR/009/3355 (See supplementary file).

## Results

**Participant´s characteristics:** during the study period, a total of 80 participants were recruited. Only 63 of the participants (60.3% females and 39.7% males) had their HIV-1 pol region successfully sequenced and analyzed. The mean age of the study participants was 31.14 (1-66 years) and the mean viral load was 4.41 (2.97-6.05) log_10_ copies/ml. There were no significant differences in sex, age, ART regimen line and plasma viral loads verses HIV-1 subtypes. Annex 1 and Table 1 show the demographic characteristics of the study participants and study outcomes verses HIV-1 subtypes and recombinants respectively.

**Table 1:** study outcomes verses HIV-1 subtypes and recombinants

HIV-1 subtypes, no % of individuals					
Characteristic	Overall (n=63)	Pure subtypes (n=54)	Recombinants (n=9)	P	χ2
**Age**					
0-14 (Pediatrics)	16 (25.4)	15 (27.8)	8 (88.9)		
15+ (Adults)	47 (74.6)	39 (72.2)	1 (11.1)	0.4	1.13
**Gender**					
Female	38 (60.3)	30 (55.6)	8 (88.9)		
Male	25 (39.7)	24 (44.4)	1 (11.1)	0.75	3.58
**ART Regimen**					
First Line	52 (82.5)	43 (79.6)	9 (100.0)		
Second Line	12 (17.5)	12 (20.4)	0 (0.0)	1	1
HIV-1 load, median log10 copies/ml	4.27	4.28	4.16	0.91	50.75

N is the total number of participants; study outcomes such as age, sex and treatment line regimen were compared with the number of study individuals having pure subtypes and recombinant forms

**Figure 1:**
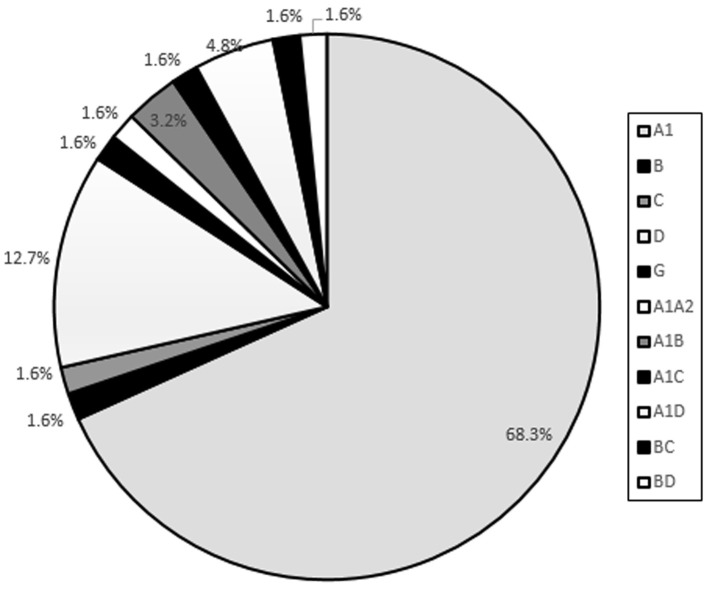
HIV-1 subtypes and recombinants identified in the present study conducted in Teso region of Western Kenya in 2019

**HIV-1 subtypes:** analysis of the 63 samples revealed 5 pure subtypes and 6 recombinants. The pure subtypes identified were (A1, 68.3%; D, 12.7%; B, C and G each 1.6%). The recombinants on the other hand were (A1-D, 4.8%; A1-B, 3.2%, A1-A2, A1-C, BC and BD each 1.6%). Figure 1 shows the distribution of pure and recombinant HIV-1 subtypes identified in the study.

**Figure 2:**
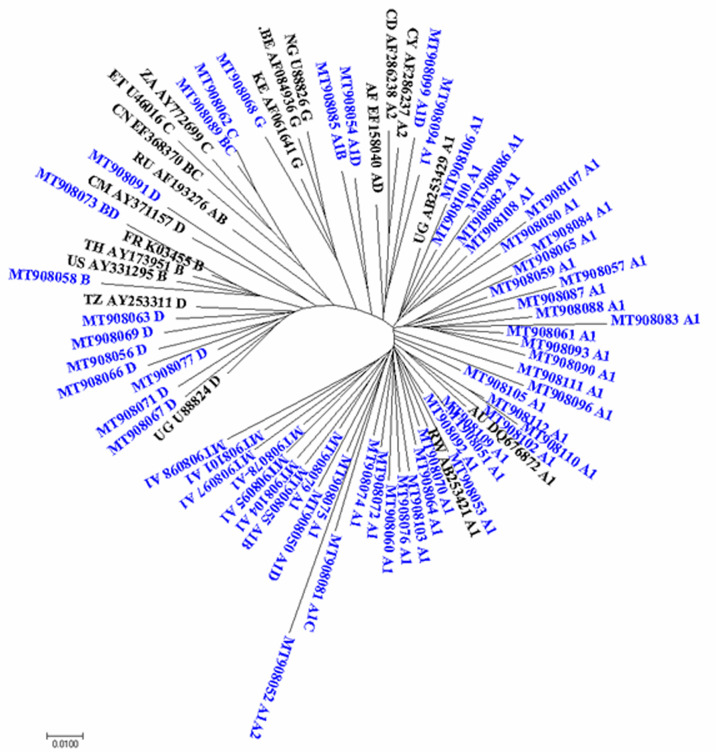
phylogenetic relationship of pure subtypes and recombinants HIV-1 sequences identified in the study

**Phylogenetic analysis:** HIV-1 sequences obtained from the study were denoted with (blue) Genbank accession numbers: MT908050-MT9080112 while reference sequences denoted with (black) accession numbers. An evolutionary distance of 0.01 nucleotides per position in the sequence is indicated in the scale bar (1% differences between two sequences). Figure 2 shows phylogenetic relationship of pure subtypes and recombinants HIV-1 sequences identified in the study. Phylogenetic classification of these sequences showed differing length of branches and clustering patterns of closely related and distantly unrelated sequences. It was observed that the recombinant forms clustered with pure subtypes for example; recombinants A1-C, A1-A2, A1-D and A1-B clustered with subtype A1 sequences and BD clustered with subtype B reference sequences from France, Thailand and United States. Subtype A1 sequences obtained from the study clustered with reference sequences from Uganda, Rwanda and Australia. The subtype D sequences clustered with reference sequences from Tanzania and Uganda. Sequence MT908091 did not cluster with other subtype D sequences instead it clustered with subtype D reference sequence from Cameroon. Subtype C clustered with recombinant BC. Subtype G sequences clustered with reference sequences from Nigeria and Belgium.

## Discussion

This study was focused on determining the distribution of HIV-1 and phylogenetic relationship of subtypes circulating in Teso region of Western Kenya. This region has two border points with Uganda. Of the 80 samples, 63 were successfully sequenced. Seventeen samples were not proceeded to sequencing due to undetectable complementary DNA during gel electrophoresis. This outcome is similar to a study carried out in Northern Eastern South Africa which had 30% of its samples not proceeding to sequencing due to undetectable complementary DNA during gel electrophoresis [31]. Participants with greater than 1000 copies/ml of blood were recruited in the study due to the success of sequencing attributed to these high viral load copies [32]. Participants showed that the proportion of females who were recruited in this study were more than males. Females were more because the HIV prevalence in the country among women is 6.6%, twice that in men at 3.1% [33]. Men on the other hand are more probably tested for HIV when they present themselves to health facilities with poor health conditions and/or present themselves at severe stages of the disease [34]. The mean age of the study participants from the study was 31.14 years, a similar study conducted in Northern Tanzania identified a mean age of 31.7 years among the study subjects which is similar to the mean age in this study [35]. It was observed that a large proportion of the adults (15+ years) as compared to pediatrics (0-14 years) were recruited in this study. These results are supported by previous findings from the Kenya AIDS response progress report 2018 which reported that the number of adults living with HIV were more than pediatrics [33]. In addition, participants of 0-14 years are only one age group while those of 15+ years are four age groups. Most of the study participants were on first line regimen in contrast to those on second line regimen. This is attributed to the fact that all HIV positive individuals are initiated on first line regimen and only switched to second line regimen after confirmation of treatment failure [27]. Further analysis of the variables showed no significant statistical difference of HIV-1 subtypes verses other variables including age, gender, ART regimen line and viral load copies with P>0.05.

The current study identified five pure subtypes and six recombinant forms. Subtype A1 was the most predominant followed by D, and C, B, G the least. These findings are in concordance with other studies conducted in Kenya which reported subtype A1 to be the most predominant in Kenya [20,21,23,24,36]. The past study conducted a decade ago identified similar subtypes A1, B, C, D and G that were also identified in the current study. However, the current study did not identify A2 which was identified in the previous study [20]. Other previous studies identified subtypes A1, C and D which were also identified in the present study but did not report subtype B [21-24]. Another study conducted in Central Kenya did not identify subtype G which was identified in the current study [24]. A past study carried out a decade ago identified only one isolate of subtype B as well as the current study [20]. This means that subtype B is less transmissible compared to other subtypes. It was identified that subtype frequencies were dependent on geographical regions for example a study conducted in Nairobi identified 13 (9%) C and 24 (17%) D while the present study identified 1 (1.6%) C and 8 (12.7%) D [23]. This shows that if a larger sample size was used in this study the subtypes identified will be slightly more than those identified in past studies. The predominance of subtype A1 in the study population is mainly because of early introduction of subtype A1 as reported by its predominance in all previous genetic diversity studies, multiple introduction of subtype A1 into the study population from other countries/regions of Kenya, faster diversification and its high transmissibility as compared to other subtypes [10,20,24,37]. Few isolates of subtypes B, C, D and G were identified in the present study this could be attributed to the fact that they originated from different countries and they might have less patterns of transmission [4].

The current study also identified circulating recombinants of HIV-1. The predominant recombinant being A1-D followed by A1B and each of A1-A2, A1-C, BC and BD in that order. These finding are in agreement with past studies which identified A1D as the predominant recombinant [20-23]. A previous study identified subtype A1A2 which was also identified in this study [21]. Past studies have also identified recombinants A2C, A2D and AG [21-23] which were not identified in the present study. The current study identified recombinants A1B and BD that were not reported in the past studies in Kenya [20,21,23,24,36]. The certainty of the transmission patterns of A1B and BD recombinants is unknown since the present study was the first to point out in the Kenyan population. The introduction of subtype B in the Kenyan population which has its origin in Europe, Japan, America, Thailand and Australia over a decade ago as reported by previous studies due to migration of people from this countries into Kenya [20] has resulted to recombination with the existing subtypes (A1 and D) resulting to emergence of new recombinants A1B and BD which have never been reported in Kenya. The emergence of recombinants A1-B, and BD and continued presence of A1-A2, A1-C, A1-D and BC indicates that the human body is more suited for the survival of recombinants in this period of scale up of antiretroviral therapy [23]. The recombinants might have also arose from transmission of viruses that already recombined from HIV-1 dually infected persons in this study population or from other regions [23]. In future, it´s suspected there might be increased viral diversity as several subtypes, sub-subtypes and recombinants are introduced in the population as seen by the number of subtypes and recombinants increasing steadily over the years. For example four different subtypes and recombinants were identified over a decade ago [21] and this study identified five different subtypes and six different recombinants.

Phylogenetic analysis of the sequences from this study showed close clustering of A1, C, D, G and their recombinants with global reference sequences from Kenya, Rwanda, Uganda, Australia, Tanzania, Cameroon, Nigeria, France and United States as per the Los Alamos HIV database. A number of sequences were derived from the same ancestor and had similar branch lengths showing that these sequences were phylogenetically closely related despite coming from different countries. These similarities suggest restricted evolutionary trend and virus transmission within the population across the country. This shows that movement patterns result to introduction of new viral strains from those countries into the study population. The results of this study show that the pol gene has got enough intrinsic genetic variation that allow phylogenetic analysis by the reconstruction of transmission histories [38]. Divergence of the virus may arise due to large differences between the virus from transmission events and convergence due to transmission of similar sets of mutations [39].

## Conclusion

An increased diversity of HIV-1 subtypes and recombinants reported in this study as compared to the previous studies in Kenya poses a threat. This was because the emergence of a certain subtype or recombinant have differing outcomes on rates of transmission, disease progression and development of drug resistance. Furthermore, this study reports recombinants A1B and BD that have never been reported in the previous studies in Kenya. The new recombinants indicate that there could be new subtypes being introduced and circulating among the Kenyan population due to cross-border movement of people from different regions of the world. This calls for continuous surveillance and monitoring of HIV-1 subtypes in the country that will aid in drug design and development for effective management of HIV infection.

### What is known about this topic

- HIV-1 subtypes are geographically distributed and movement patterns influence introduction of new subtypes in a given region;
- HIV-1 genetic variability is an obstacle in treatment of HIV and development of effective drugs.

### What this study adds

- The findings from the study show increased diversity of HIV-1 subtypes and recombinants that pose challenges in the management of HIV infection;
- The outcomes add to the existing knowledge of HIV-1 subtypes for instance the identification of new recombinants that have never been identified previously;
- There is a relationship between the new identified recombinants with the existing subtypes and recombinants and this will aid in the design and development of drugs.

## Annex

**Annex 1**: study participant's demographic characteristics (PDF-159Kb)
